# Repetitive elements as a transcriptomic marker of aging: Evidence in multiple datasets and models

**DOI:** 10.1111/acel.13167

**Published:** 2020-06-05

**Authors:** Thomas J. LaRocca, Alyssa N. Cavalier, Devin Wahl

**Affiliations:** ^1^ Department of Health and Exercise Science Center for Healthy Aging Colorado State University Fort Collins CO USA

**Keywords:** aging, *Caenorhabditis elegans*, human, repetitive elements, transcriptomics, transposable elements

## Abstract

Transcriptomic markers of aging can be useful for studying age‐related processes and diseases. However, noncoding repetitive element (RE) transcripts, which may play an important role in aging, are commonly overlooked in transcriptome studies—and their potential as a transcriptomic marker of aging has not been evaluated. Here, we used multiple RNA‐seq datasets generated from human samples and *Caenorhabditis elegans* and found that most RE transcripts (a) accumulate progressively with aging; (b) can be used to accurately predict age; and (c) may be a good marker of biological age. The strong RE/aging correlations we observed are consistent with growing evidence that RE transcripts contribute directly to aging and disease.

## Graphical abstract

Repetitive element transcripts may accumulate progressively with aging and, therefore, be a good transcriptomic marker of age/aging. We analyze multiple RNA‐seq datasets from humans and model organisms and show that repetitive element transcripts may: (a) be used to predict age; and (b) be a marker of biological aging.

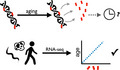

## 1 INTRODUCTION

Older age is the primary risk factor for disability and chronic diseases, and as a result, there is significant interest in identifying mechanisms and markers of aging (Partridge, Deelen, & Slagboom, 2018). One powerful way to examine biological signatures of aging is to profile differential expression of genes and signaling networks that may be involved in the aging process (e.g., by RNA‐seq). Using this approach, some groups have studied the transcriptional landscape of aging in blood (Peters et al., 2015) and used machine learning to predict age based on the transcriptome (Fleischer et al., 2018). However, many analyses have focused on known/coding genes and overlooked noncoding genetic material (the majority of the genome).

One particularly large and often‐ignored fraction of the human genome (>60%) is composed of repetitive elements (RE). These include: types 1 and 2 transposons (retrotransposons and DNA transposons, respectively), some of which can self‐copy and reinsert into new locations; terminal repeats (at the ends of retrotransposons); and tandem repeats (including sequences common to centromeres, chromatin, and other structured genome regions) (Cordaux & Batzer, 2009). Most RE are chromatinized and suppressed, but certain retrotransposons remain active in humans and may be involved in aging (Kreiling et al., 2017). Indeed, studies in mice and other model organisms have shown that active/transposable RE, in particular, contribute to the aging process (Chen, Zheng, Xiao, & Zheng, 2016; De Cecco et al., 2019; Van Meter et al., 2014; Wood et al., 2016), although most evidence points to RE activation later in life (e.g., in senescence). The potential for RE *in general* to serve as a transcriptomic marker of aging has not been investigated (especially in humans), but we and others have reported a generic accumulation of RE transcripts (i.e., not only active RE) in age‐related neurodegenerative processes and diseases (Guo et al., 2018; LaRocca, Mariani, Watkins, & Link, 2019; Li, Jin, Prazak, Hammell, & Dubnau, 2012; Saldi, Gonzales, LaRocca, & Link, 2019). Evidence also indicates that chromatin maintenance declines with aging, which could increase general transcriptional accessibility of RE (Field & Adams, 2017). As such, age‐related changes in global RE transcript levels could be a good transcriptomic/mechanistic marker of aging.

## 2 RESULTS AND DISCUSSION

**Figure 1:**
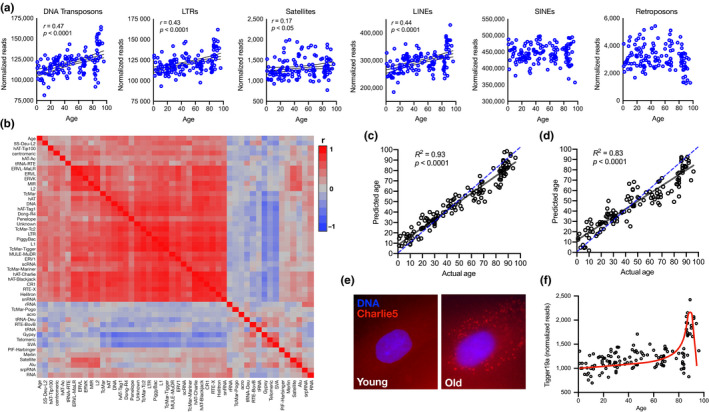
Age‐related RE transcript accumulation predicts donor age in human fibroblasts. (a) Correlations between age and different types of RE transcripts. (b) Heat map showing correlations among RE transcript families and age. (c) Linear regression predicting actual age based on individual RE transcript counts (blue dashed line represents perfect correlation for predicted age with true age). (d) Linear regression predicting actual age based on the top 1,200 genes most differentially expressed with aging. (e) Fluorescence in situ hybridization confirmation of the Charlie5 RE transcript in young versus old human fibroblasts. (f) Exponential pattern of individual RE transcript levels with age in human fibroblasts (e.g., Tigger19a transposon, the RE most highly correlated with age). All bioinformatics analyses performed on RNA‐seq data from cultured dermal fibroblasts of 133 healthy human subjects, aged 1–94 years.

To determine whether RE transcripts increase globally with aging, we first examined a total RNA‐seq dataset generated from dermal fibroblasts of 133 healthy subjects aged 1–94 years (Fleischer et al., 2018). Using RepEnrich, an established bioinformatics pipeline (Criscione, Zhang, Thompson, Sedivy, & Neretti, 2014), we analyzed RE as previously described (LaRocca et al., 2019) and found that transcripts from most major types of RE increased with aging (Figure 1a). Some of these correlations were stronger when we subdivided RE into families, almost all of which were positively related to donor age (Figure 1b). Interestingly, a subset of RE families clustered/correlated more strongly with age; we did not observe any particular pattern among these elements, but we did note that many also correlated among themselves, further supporting the idea that global RE expression increases with aging.

Could a global increase in RE transcripts be a good transcriptomic marker of age/aging? To investigate this possibility, we examined individual RE transcripts more closely. We found that >60% of these correlated even more strongly with age than RE by class/family, so we asked if we could predict age from these individual transcripts. In the original study described above (Fleischer et al., 2018), the authors developed a machine learning algorithm to predict age from the standard transcriptome (genes and noncoding RNAs). However, using only RE transcripts and linear regression, we were able to predict age more accurately (*R* ^2^ = 0.93, Figure 1c). These results extend significantly on previous findings. Others have reported transcriptome signatures of aging in humans (Fleischer et al., 2018; Peters et al., 2015) and model organisms (Rangaraju et al., 2015; Tarkhov et al., 2019), but none have estimated age so closely (most *R* ^2^ ≃ 0.6–0.8). This could be because a transcriptome feature that changes consistently and in the same direction is a better marker of aging than gene expression patterns, which may increase and/or decrease with age‐related processes. Consistent with this idea, we found that thousands of genes were differentially expressed with aging in this same dataset (Figure S1), but that even when we used our same regression approach to predict age based on the genes most different with aging, we could only achieve *R* ^2^ = 0.83 (Figure 1d).

Importantly, analyses of RE in RNA‐seq data are inherently challenging and artifact‐prone because of the many RE copies and their various locations in the genome. Different programs use a variety of computational approaches to address these challenges (Bourque et al., 2018; Kaul, Morales, Sartor, Belancio, & Deininger, 2020). Therefore, we repeated our above analyses using TEtranscripts and SQuIRE, two other established pipelines for detecting RE in RNA‐seq data (Jin, Tam, Paniagua, & Hammell, 2015; Yang, Ardeljan, Pacyna, Payer, & Burns, 2019). In support of our findings, we achieved very similar results with both of these programs (Figure S1). Still, to further confirm these computational analyses, we obtained several of the same bio‐banked fibroblasts on which these RNA‐seq data are based, and we performed RNA fluorescence in situ hybridization (RNA‐FISH) for the Charlie5 transcript (a DNA transposon that is not reportedly active but correlated strongly with age and is large enough for FISH). We found very little Charlie5 FISH signal in young fibroblasts, but a marked accumulation of this RE transcript in old cells (Figure 1e). Interestingly, we also noted that many age‐related increases in individual RE transcripts could be fit to an exponential pattern (Figure 1f). This may be an important observation, because markers of *biological age* (cellular/organismal health versus chronological age in years) might be expected follow a nonlinear trajectory reflective of mortality risk with aging (e.g., exponential or Gompertz models) (Finch & Crimmins, 2016).

**Figure 2:**
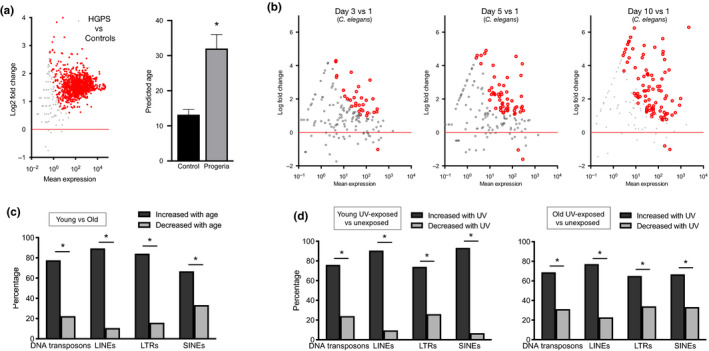
RE transcripts may be a transcriptomic marker of biological age/aging. (a) MA plot showing progeria‐related increases in RE transcripts (red data points FDR < 0.1) and age predictions based on RE expression in fibroblasts from age‐matched controls and Hutchinson–Gilford progeria patients (*n* = 10 per group, *p < 0.01 versus control). (b) MA plots showing progressive increase in RE transcripts with aging in *C. elegans* (*n* = 3 per group, red data points FDR < 0.1). (c) Percentage of RE transcripts by major type increased and decreased in human fibroblasts from older (aged 60–67) versus young (aged 18–25) donors (n = 9 per group, *p < .05). (d) Percentage of RE transcripts by major type increased or decreased with UV exposure in human fibroblasts from younger and older donors (*n* = 9 per group, *p < .05).

Future studies are needed to determine whether age‐related RE transcript increases truly reflect biological age. However, to provide initial insight, we examined RNA‐seq data on fibroblasts from patients with Hutchinson–Gilford progeria syndrome (HGPS, a premature aging syndrome) in a secondary dataset from the study described above. Using the RepEnrich pipeline, which was the most sensitive to RE/age trends in our first analyses, we found a marked increase in RE transcripts with HGPS (Figure 2a), and our regression model also predicted these progeria patients to be 3 times older than age‐matched controls. This finding suggests that RE expression may reflect biological age. However, confirming this will require additional/future testing. No other *total* RNA‐seq datasets on aging in human fibroblasts were available, and we note that RNA‐seq data may be influenced by many technical factors (e.g., library preparation methods), so different regression/prediction models may be needed for different datasets. In the context of HGPS specifically, our results could also be related to disease‐associated chromatin defects. Therefore, we next asked if similar age‐related RE increases are observable in classic organismal models of aging. We analyzed an RNA‐seq dataset on aging in *C. elegans* (Rangaraju et al., 2015) and found a progressive, age‐related increase in RE transcripts (Figure 2b), and we confirmed that this observation in a nonhuman model was reproducible using TEtranscripts (Figure S1). Taken together, these results suggest that global dysregulation of RE transcripts may indeed be a clue to a conserved mechanism of aging.

Finally, to further substantiate our findings and explore the possibility that RE expression might reflect biological age, we analyzed RE transcripts in an additional human fibroblast dataset including cells from the buttock and shoulder (unexposed versus sun‐exposed) of healthy younger and older adults (Kaisers et al., 2017) using RepEnrich. These data are based on Poly(A)‐selected libraries, which may not capture many RE. Even so, consistent with our earlier results, we found that transcripts from the major RE types were increased in fibroblasts from older versus young subjects (Figure 2c). We also found that RE transcripts were greater in shoulder‐derived samples, and this effect was more pronounced in young adults (Figure 2d). Interestingly, the authors of the original study did not find strong effects of aging on the standard transcriptome. Thus, these data further demonstrate that global, age‐related increases in RE transcripts occur consistently, are reproducibly detectable in different datasets, and may be a more sensitive transcriptomic marker of aging than gene expression. They also provide additional evidence that RE transcript accumulation could reflect biological age (i.e., because UV exposure might increase biological age but would not be expected to directly impact RE).

The present findings may be an important addition to the current understanding of RE in aging. RE de‐repression has been documented in senescent cells (Sedivy et al., 2013), and RE transcripts have been implicated in inflammation and oxidative stress (Kreiling et al., 2017), two key contributors to aging and disease. The mechanisms by which RE transcripts stimulate these processes are unclear, but could involve activation of innate immune responses. For example, recent reports show that specific transposable RE (e.g., LINEs) become active with aging and promote senescence‐associated inflammation, and suppressing LINE activity has anti‐aging effects (De Cecco et al., 2019). This is an important area of research, as active RE may be particularly damaging to the cell—and bioinformatics pipelines that detect them in RNA‐seq data may be useful in this context. However, in our data we noted that active/transposable RE (e.g., L1Hs) did not correlate any better than other RE with age. Furthermore, the increased expression of presumedly inactive RE that we observed (e.g., Charlie5) points to a more basic dysregulation of RE by the cell. Our data suggest that a progressive, global dysregulation of RE transcripts (i.e., not only active/transposable RE in senescence) could be: (a) a clue to an important mechanism and/or consequence of aging; (b) a marker of biological age; and (c) a novel therapeutic target. In support of these ideas, we recently reported that RE transcript accumulation is associated with age‐related neurodegenerative diseases in patient brains, and that suppressing cellular immune sensors reduces neuroinflammatory signaling downstream of RE (LaRocca et al., 2019; Saldi et al., 2019). Future investigations are needed to determine how global RE transcript increases may contribute to aging per se, and whether this might be targeted *in vivo*.

One possible, therapeutically relevant explanation for our findings is that age‐related chromatin changes may facilitate RE transcription. Indeed, aging is associated with depletion of chromatin‐organizing histone proteins and reduced epigenetic/histone maintenance (Field & Adams, 2017). Moreover, suppressing age‐related chromatin changes increases lifespan in model organisms, and many anti‐aging interventions are associated with lasting chromatin changes. An alternative possibility is that RE transcript accumulation during aging results from reduced transcript degradation, which would be consistent with the observation that most cellular recycling pathways (e.g., autophagy) decline with aging. This too may be a promising area for future study.

Most broadly, our findings may be useful to those interested in transcriptomic markers of age/aging. Classic genomic markers like telomere length and the epigenetic clock have been useful for studying age and factors that accelerate aging in many settings. Whether RE transcripts are a *bona fide* transcriptomic biomarker of biological aging remains to be determined, and it may be necessary to develop a measurement of RE transcripts that is reproducible across different studies (e.g., total RE transcript abundance) to test this idea. In any case, based on existing data and our current findings in multiple datasets and models, we speculate that increased RE expression will be implicated in other models and diseases of aging in the near future.

## 3 EXPERIMENTAL PROCEDURES

### 3.1 RNA‐seq datasets and availability

The data that support the findings of this study (RNA‐seq datasets) are available in the Gene Expression Omnibus (GEO), accession numbers GSE113957 (human fibroblasts) and GSE63528 (*C. elegans*), and at ArrayExpress (human fibroblasts), accession number E‐MTAB‐4652.

### 3.2 Bioinformatics analyses

RE transcripts were analyzed in all datasets using the RepEnrich algorithm (Criscione et al., 2014) as previously described (LaRocca et al., 2019) and/or the TEtranscripts and SQuIRE programs (Jin et al., 2015; Yang et al., 2019) to compare findings. Briefly, reads were trimmed and quality filtered with the *fastp* program, then aligned to the genome (hg38 *Homo sapiens *or ce10 *C. elegans*) using Bowtie (for RepEnrich) or the STAR aligner (for TEtranscripts and SQuIRE). RE transcripts were then quantified with RepEnrich, TEtranscripts or SQuIRE, which are Python‐based programs that use different strategies to combine counts from uniquely mapping and multi‐mapping RE reads and then generate counts for individual RE, as well as RE by class and family. RE transcript counts were normalized to library size for statistical modeling or analyzed for differential expression as described below. To compare genes versus RE for age predictions, the 1,200 genes most significantly different with aging (similar to the number of RE detected by the three pipelines) were identified by mapping gene expression/counts in RNA‐seq data from the ten oldest and youngest adult fibroblast donors using STAR and differential expression analyses as described below.

### 3.3 Human cell culture and fluorescence in situ hybridization

Human dermal fibroblasts were obtained from the Coriell Institute and were selected to match RNA‐seq data/samples (Fleischer et al., 2018) from young (GM04501, GM03377, aged 19) and old (AG09843, AG11748, aged 86) adult donors. Cells were grown for 2 passages in fibroblast growth medium in a humidified incubator (5% CO_2_, 37°C) and then fixed for fluorescence in situ hybridization. RNA‐FISH probes for the repetitive element Charlie5 were designed using the LGC Stellaris Probe Designer and used according to manufacturer's instructions (LaRocca et al., 2019), and FISH signals were imaged on an EVOS M7000 inverted fluorescence microscope (Invitrogen) at 100× magnification.

### 3.4 Statistical analyses

Bivariate correlations, heat maps, and linear regression models of RE transcripts (counts normalized to library size) and age in human fibroblasts were generated using JMP Pro software. Differential expression of genes and RE transcripts in fibroblasts and *C. elegans *was analyzed with Deseq2 as previously reported using sample‐specific size factors to account for differences in library size in RE analyses (LaRocca et al., 2019). Chi‐square analyses of increased versus decreased RE types in human fibroblasts were performed using Prism/GraphPad software.

## CONFLICT OF INTEREST

The authors declare that they have no conflicts of interest.

## AUTHOR CONTRIBUTIONS

TJL conceived and designed the study. TJL, ANC, and DW collected and analyzed the data. TJL drafted the manuscript with input and assistance from all authors, and TJL, ANC, and DW edited the final manuscript.

## Supporting information

Figure S1

## Data Availability

The data that support the findings of this study (RNA‐seq datasets) are available in the Gene Expression Omnibus (GEO), accession numbers GSE113957 (human fibroblasts) and GSE63528 (*C. elegans*), and at ArrayExpress (human fibroblasts), accession number E‐MTAB‐4652.
