# Supplementary figures and images for: Repetitive elements as a transcriptomic marker of aging: Evidence in multiple datasets and models
Source: Aging Cell. 2020 Jun 5;19(7):e13167. doi: 10.1111/acel.13167 (PMC7412685; doi:10.1111/acel.13167)

Supporting Information Figure S1

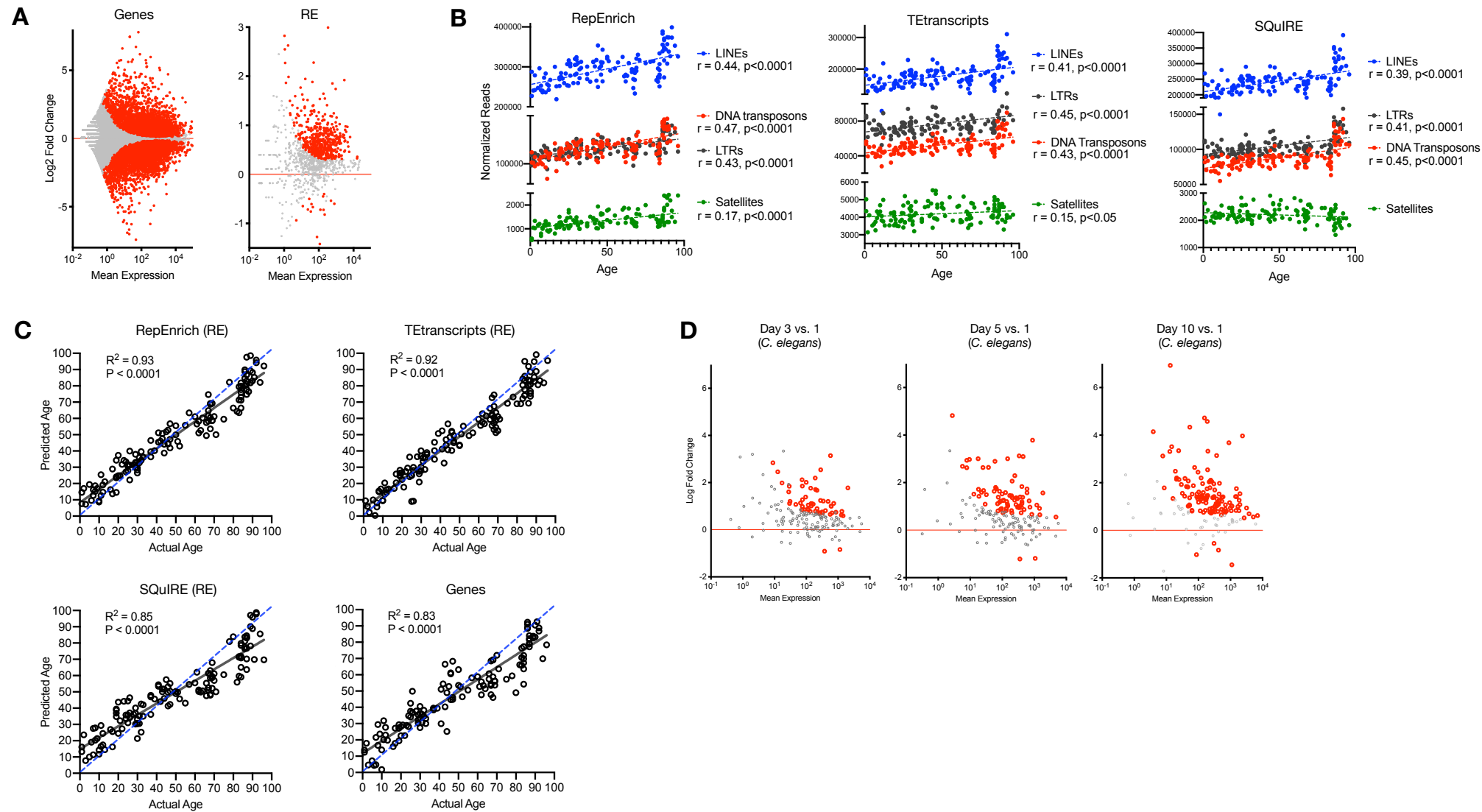

Supplement: Supplementary file 1 — Figure S1 [file ACEL-19-e13167-s001.pdf]
